# Cancer Patient Tissueoid with Self‐Homing Nano‐Targeting of Metabolic Inhibitor

**DOI:** 10.1002/advs.202102640

**Published:** 2021-10-18

**Authors:** Hyo‐Jin Yoon, Young Shin Chung, Yong Jae Lee, Seung Eun Yu, Sewoom Baek, Hye‐Seon Kim, Sang Wun Kim, Jung‐Yun Lee, Sunghoon Kim, Hak‐Joon Sung

**Affiliations:** ^1^ Department of Medical Engineering Yonsei University College of Medicine Seoul 03722 Republic of Korea; ^2^ Department of Obstetrics and Gynecology Institution of Women's Life Medical Science Severance Hospital Yonsei University College of Medicine Seoul 03722 Republic of Korea

**Keywords:** cancer cell‐derived nanovesicles, ovarian cancer, patient‐specific treatments, self‐homing nano‐targeting, tissueoid

## Abstract

The current paradigm of cancer medicine focuses on patient‐ and/or cancer‐specific treatments, which has led to continuous progress in the development of patient representatives (e.g., organoids) and cancer‐targeting carriers for drug screening. As breakthrough concepts, i) living cancer tissues convey intact profiles of patient‐specific microenvironmental signatures. ii) The growth mechanisms of cancer mass with intense cell‐cell interactions can be harnessed to develop self‐homing nano‐targeting by using cancer cell‐derived nanovesicles (CaNVs). Hence, a tissueoid model of ovarian cancer (OC) is developed by culturing OC patient tissues in a 3D gel chip, whose microchannel networks enable perfusion to maintain tissue viability. A novel model of systemic cancer responses is approached by xenografting OC tissueoids into ischaemic hindlimbs in nude mice. CaNVs are produced to carry general chemotherapeutics or new drugs under pre/clinical studies that target the BRCA mutation or energy metabolism, thereby increasing the test scope. This pioneer study cross‐validates drug responses from the OC clinic, tissueoid, and animal model by demonstrating the alignment of results in drug type‐specific efficiency, BRCA mutation‐dependent drug efficiency, and metabolism inhibition‐based anti‐cancer effects. Hence, this study provides a directional foundation to accelerate the discovery of patient‐specific drugs with CaNV application towards future precision medicine.

## Graphical abstract

A microchannel chip enables direct perfusion culture of ovarian cancer tissues from patients. Consequently, drug efficacies are cross‐validated with animal and clinical outcomes towards patient‐specific medicine. Considering spontaneous cancer cell aggregation upon propagation, cancer cell‐derived nanoparticles are produced to program self‐homing to target cancer sites. Loading of metabolic inhibitors to the particles represents a “trojan horse”‐like strategy.

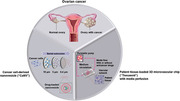

## 1 Introduction

Recent advances in biotechnologies have been integrated with precision medicine. As a major outcome, the paradigm of anti‐cancer drug treatment has become to focus on patient‐specific selection and cancer‐specific targeting.^[^ ^1^ ^]^ A common goal of current outstanding technologies is to expedite clinical success by cross‐validating the safety, efficacy, and efficiency of anti‐cancer drugs among in vitro, in vivo, and clinical studies. To this end, extensive efforts have been made to develop artificial models (e.g., cell and organoid) of patient representatives^[^ ^2^ ^]^ for drug screening as a potential platform for cross‐validation with in vivo responses through implantation.^[^ ^3^ ^]^ Despite the promising potential of cells and organoids to exhibit patient‐specific genetic and cancer characteristics, an unmet need is to include microenvironmental signatures, such as extracellular matrix (ECM) components, vasculature, cancer‐associated cells, as well as physical (e.g., stiffness), chemical (e.g., paracrine deposit in ECM), and mechanical (e.g., blood flow) properties. Since it is impossible to test anti‐cancer drugs directly in patients and thus skip preclinical models, the use of living tissues from cancer patients is suggested to convey intact patient‐specific profiles, not only of genetic and cancer characteristics, but also microenvironmental signatures, representing a potential solution to the existing unmet need.

On the other hand, the concept of cancer‐specific targeting has been approached by exploiting specific receptor‐ligand interactions between drug carriers and cancer cells.^[^ ^4^ ^]^ As it is almost impossible to find an exclusively expressed molecule on target cancer cells,^[^ ^5^ ^]^ the consensus on the efficiency of this approach is tarnished. Nonetheless, the scale of the receptor‐ligand interaction can be amplified to cell‐cell interactions observed in cancer mass growth with cell stacking,^[^ ^6^ ^]^ suggesting that cancer cells are capable of targeting their own type of cells. Therefore, the breakthrough strategy of self‐homing nano‐targeting can be applied. The reciprocal interaction of cell membranes is a key mediator of cancer cell‐cell interactions, whose function can be potentiated using cancer cell‐derived nanovesicles (CaNV), owing to i) the same membrane characteristics as target cancer cells; ii) inanimation to prevent living cell‐mediated side effects; iii) effective systemic delivery similar to exosomes,^[^ ^7^ ^]^ as revealed by another group;^[^ ^8^ ^]^ and iv) more efficient mass production and purification^[^ ^9^ ^]^ compared to exosomes.^[^ ^10^ ^]^ Moreover, the loading of anti‐cancer drugs into CaNV represents a “Trojan horse” strategy to inhibit cancer action synergistically with the self‐homing nano‐targeting approach.

Ovarian cancer (OC) serves as a cancer model in this study, due not only to the high mortality rate,^[^ ^11^ ^]^ but also the limited treatment options, making it a significant global issue. A wider spectrum of drug options could save patients with acquired resistance to chemotherapy or in the advanced stages of cancer progression (III or IV).^[^ ^12^ ^]^ Hence, new drugs must be tested, despite their clinical efficiency not yet being proven. One such example is the cancer energy metabolism‐based drug (MB‐Drug), as the regulation of cancer cell metabolism is a promising function of a drug candidate.^[^ ^13^ ^]^ Another drug option is Olaparib (Ola), which has recently been approved as a targeted treatment to the BRCA‐mutation (mut) group at advanced OC stages, as it inhibits DNA repair by poly (ADP‐ribose) polymerase (PARP) enzymes, thereby inducing death of BRAC‐mut OC cells with incremental damage to the DNA.^[^ ^14^ ^]^

To answer this need, this study introduces the novel concept of an implantable OC tissueoid capable of culturing OC patient tissues in vitro and in vivo. Drug type‐specific efficiency, BRCA mutation‐dependent drug efficiency, and metabolism inhibition‐based anti‐cancer effects were tested. CaNVs were produced to deliver general chemotherapeutics, as well as the two aforementioned drugs following the concept of Trojan horse. Based on more than 100 patient records, the cross‐validation among in vitro, in vivo, and clinical studies was carried out as the first step of a paradigm shift in the field of new drug development.

## 2 Results

### 2.1 OC Patient Tissues

During the past two years (Table S1, Supporting Information), OC tissues were collected from 104 patients through biopsy for diagnosis or surgery. Two samples of normal ovary tissue were also obtained from patients with no evidence of disease, based on the final pathologic reports. The median age of patients at diagnosis was 57.5 years in a range of 37.0–78.0 years. Patient records showed high levels (median 1735.6 U mL^−1^) of the cancer antigen (CA)‐125 as a clinical OC marker (normal upper limit: 35 U mL^−1^) at diagnosis. According to the International Federation of Gynaecology and Obstetrics (FIGO) stage^[^ ^15^ ^]^ (i.e., malignant stage based on cancer size and progress), all patients were diagnosed with the advanced stage (III: n = 45 or IV: n = 59). Most patients (n = 86, 82.7%) were diagnosed with the high‐grade serous carcinoma subtype after histological analysis of tumor tissues post‐resection surgery. All patients (100%) received paclitaxel (PTX) and carboplatin (CBP) treatment before surgery and/or after surgery as a standard chemotherapy regimen. Debulking Surgery (DS) was also conducted in all patients (100%) to remove tumor mass to the furthest extent possible before or in‐between PTX + CBP treatment. To examine the remaining degree of tumor mass, a biopsy was performed post DS, which provided the corresponding OC tissue samples for this study.

### 2.2 OC Tissueoid as a New Drug Testing Model

**Figure 1:**
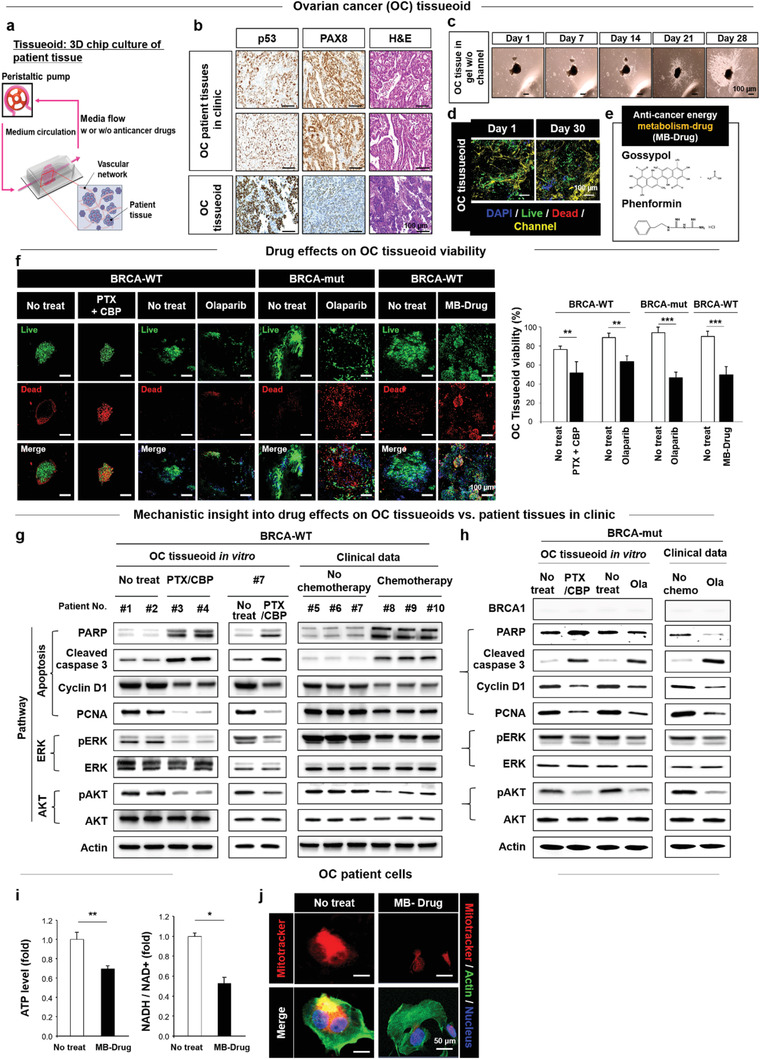
Clinically matched drug responses of OC tissueoid with the introduction of anti‐cancer metabolic (MB)‐Drug. a) The tissueoid is defined as a culture of patient tissues in a 3D chip under perfusion of media (+/− drug) into microchannel (vascular) networks at a flow rate of 20 µL min^−1^. b) OC marker expression (PAX8 and p53) and histology of tissueoids were matched with those of tissue samples from high‐grade serious OC patients in‐clinic, analyzed by optical examination with immunohistology and H&E staining. c) The gelatin gel allows the progressive cell invasion from OC patient tissues during 28‐day culture post embedding into the gel, as observed by optical imaging. d) The viability of the OC tissueoid was maintained during 30 days of perfusion culturing, as determined by confocal imaging. e) Gossypol and phenformin were used in combination as an anti‐cancer energy metabolism (MB)‐Drug. f) To represent patient tissue [+/− BRCA mutation (mut)], drug type‐dependent viabilities of OC tissueoids were validated under treatments with PTX + CBP, Ola, or MB‐Drug by confocal imaging analysis (each group, n = 3). Clinically matched responses of OC tissueoids w/ versus w/o anti‐cancer treatment for g) BRCA‐WT and h) BRCA‐mut patients were determined with respect to the protein expression of apoptosis markers (PARP, cleaved‐caspase 3, cyclin D1, PCNA), ERK pathway markers (pERK and ERK), and AKT pathway markers (pAKT and AKT) by Western blot analyses. Anti‐cancer effects of MB‐Drug are evidenced by i) ATP levels and NADH/NAD+ in OC tissueoids by bioassays (each group, n = 3) and j) mitochondrial activity for energy production in OC patient cells by confocal fluorescence imaging. Data are presented as mean ± S.E.M. Significance was determined by Student's t‐test analysis in (f), (i). *p < 0.05, **p < 0.01, and ***p < 0.001 versus no treatment or between lined groups.

For the OC tissueoid model, pieces of patient tissue were punched and subjected to culture in a 3D gel chip, whose vascular‐mimetic microchannel network was perfused with media by using peristatic pumping (20 µL min^−1^) to maintain tissue viability (**Figure** **1a**). The preservation of patient characteristics in the OC tissueoid with regard to cancer marker expression (PAX8 and p53) and histology was confirmed by matching with tissues from high‐grade serous OC patients in‐clinic (Figure 1b). The results indicate a promising capability of the OC tissueoid to carry the marker and histological signatures from the corresponding OC patient tissue in‐clinic. When cultured in the 2D gel without channel networks, cell propagation from the OC tissueoid continued for 28 days (Figure 1c). This indicates proper stiffness and cell‐friendly composition of the gelatin gel post crosslinking by the microbial transglutaminase (mTG) reaction.^[^ ^16^ ^]^ When culture media was perfused by generating microchannel networks in the gel, the high level of OC tissueoid viability was maintained until day 30 post culture (Figure 1d).

The IC 50 concentrations of PTX (5 nm) + CBP (100 µm) were determined to reduce ≈ 50% viability of OC patient tissues with BRCA‐wild type (WT) upon co‐treatment with the two drugs in a wide range of concentrations (Figure S1a, Supporting Information). The IC 50 concentration of Ola (100 µm) was likewise determined by treating BRCA‐mut OC patient tissues (Figure S1b, Supporting Information). In the same manner, using the MB‐Drug with the chemical structures (Figure 1e), the IC 50 concentrations of Gossypol (100 µm) + Phenformin (1000 µm) were determined by co‐treating OC patient tissues (BRAC‐WT, Figure S1c, Supporting Information) or human OC (SKOV3) cell lines (Figure S1d, Supporting Information). Previous studies reported 20–40 µg mL^−1^ as an effective range of NV dosage to exert anti‐cancer and stem cell‐derived effects.^[^ ^17^ ^]^ Since CaNV was injected to each mouse every week for 8 weeks, the minimum effective dosage (20 µg mL^−1^) was applied for each injection so that the effective dosage could be accumulated to serve as a cargo of MB‐Drug efficiently. Approximately 1 × 10^4^ cells were needed to produce 1 µg of NVs, and the size of biopsied OC tissue was not consistently enough to produce the sufficient amount of patient‐derived CaNVs for ever week injection during 8 weeks. Therefore, the matching patient‐derived OC cells with each tissueoid could not be used as a source of drug‐loaded CaNVs.

These test drugs (PTX/CBP, Ola, and MB‐Drug) were administered to OC tissueoids of the BRCA‐wild type (WT) and/or BRCA‐mut groups for one week by perfusion through the channel network, which was followed by determining their corresponding viabilities (Figure 1f). Anti‐cancer effects of the Ola and MB‐Drug were observed on the BRCA‐mut and BRCA‐wt groups, respectively, while the PTX/CBP anti‐cancer effect was observed in both patient groups. These results validated the efficacy of the test drugs as the standard regimen for OC treatment (PTX/CBP),^[^ ^18^ ^]^ a PARP inhibitor (Ola),^[^ ^19^ ^]^ and an energy metabolism inhibitor (MB‐Drug).

Next, protein expression of anti‐cancer signaling factors was examined to reveal drug mechanisms of PTX/CBP or Ola on OC tissueoids of BRCA‐wt and/or BRCA‐mut groups (Figure 1g,h) one‐week post‐treatment. Among general candidates of anti‐cancer pathway, the PTX/CBP treatment on the BRCA‐wt group (Patient # 1–4 and 7) predominantly affected apoptosis signaling,^[^ ^20^ ^]^ as evidenced by a significantly enhanced expression of PARP and cleaved caspase 3 and lower expression of Cyclin D1 and PCNA, compared to the no‐treatment group (PBS vehicle control) (Figure 1g). The results were clearly matched with the clinical data from patient tissues (Patient #5–10) post PTX/CBP treatment. Further, according to the aligned results between the OC tissueoid and clinical data among the same patients under PTX/CBP treatment, significant decreases in the expression of phosph(p)‐ERK and p‐AKT indicated supportive involvement of coupling ERK and AKT pathways in the PTX/CBP action, as phosphorylation occurs when downstream signaling is activated as a result of the reaction between ERK and AKT pathways.^[^ ^21^ ^]^

OC tissueoids from the BRCA‐mut group exhibited almost no expression of BRCA 1 (Figure 1h), confirming the knock‐down of BRCA1 expression by mutation. While the Ola treatment on the BRCA‐mut group resulted in the suppression of PARP as opposed to the PTX/CBP treatment, the protein expression of cleaved caspase‐3 increased significantly, while the Cyclin D1 and PCNA expression decreased as in the PTX/CBP treatment. These results were matched with clinical data which confirmed the action of Ola as a PARP inhibitor to induce death of the cancer cells.^[^ ^22^ ^]^ The expression patterns of ERK and AKT factors in BRCA‐mut between the Ola and PTX/CBP treatment groups were aligned. Matching of results with those of the PTX/CBP treatment on BRCA‐WT and clinical results indicates a common supportive role of coupling ERK and AKT pathways in both Ola and PTX/CBP effects, regardless of BRCA mutation. The MB‐Drug treatment significantly reduced the ATP level and NADH/NAD+ ratio (Figure 1i) in OC patient cells, as supported by the marked decrease in mitochondrial activity (Figure 1j). The results confirm the anti‐energy metabolic action of the MB‐Drug to suppress cancer propagation. In summary, the alignment of results with the clinical data suggests that the OC tissueoid is a reliable platform for testing drugs aimed at achieving the ultimate goal of expediting clinical translation.

### 2.3 CaNV for Self‐Homing Nano‐Targeting

**Figure 2:**
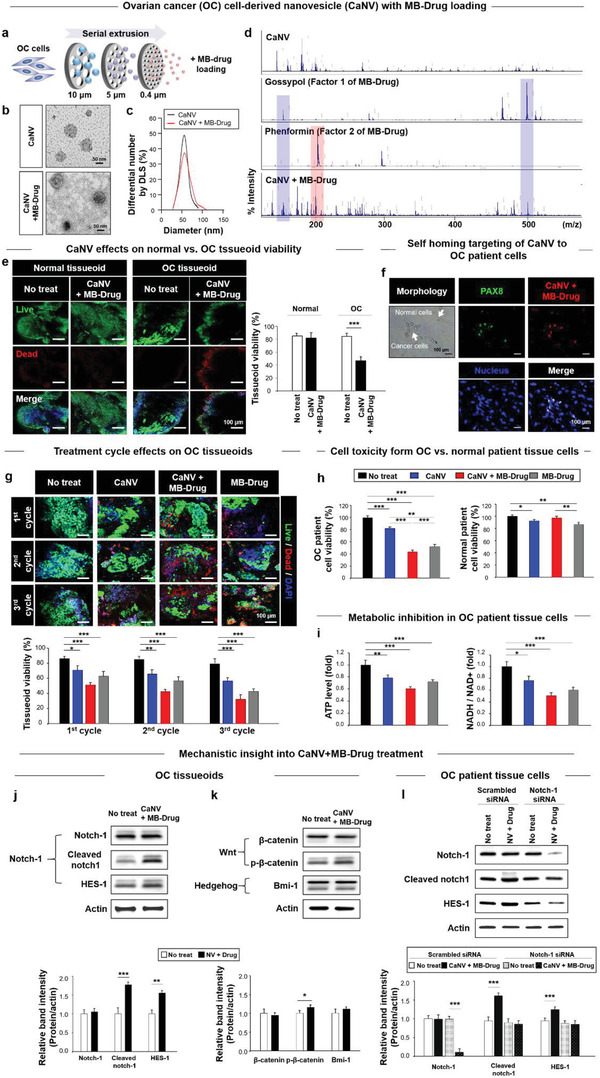
CaNVs for self‐homing nano‐targeting of MB‐Drug. a) CaNV is produced by serial filtering of OC cells with decreasing pore sizes and loading with the MB‐Drug (i.e., a combination of gossypol and phenformin) by electroporation. CaNVs+/−MB‐Drug are characterized for b) size by DLS and c) morphologies by TEM. d) The loading of MB‐Drug into CaNV was verified by analyzing each component with MALDI‐TOF/TOF. e) Cancer‐specific cytotoxic effects of test groups (each group, n = 3) were determined by analyzing the viability of normal versus OC tissueoid with confocal image analysis, which is further supported by f) OC cell (PAX8^+^)‐specific targeting and uptake of CaNV+MB‐Drug (20 µg mL^−1^) in mixture culture with normal ovarian cells by phase‐contrast and immunofluorescence imaging. g) Consequently, CaNV+MB‐Drug (20 µg mL^−1^) exerts the most efficient anti‐cancer effect among the test groups (each group, n = 3), as shown by confocal imaging. h) In alignment with the tissueoid results, the OC cell‐specific toxicity of CaNV+MB‐Drug (20 µg mL^−1^) without influencing the viability of normal ovarian cells is most apparently exhibited among the test groups upon separate treatment of both cell types. i) This is mainly due to the detrimental effects of the MB‐Drug ATP levels and NADH/NAD+ ratio in OC cells. As a mechanistic insight observed in the Western blot, j) the anti‐cancer effect of the MB‐Drug was dominantly associated with Notch‐1 signaling, as evidenced by the overexpression of downstream factors (cleaved notch‐1 and Hes‐1) in OC tissueoids upon drug treatment, despite no significant change in the Notch‐1 expression (each group, n = 3). k) In contrast, the MB‐Drug treatment does not affect protein expression of Wnt (*β*‐catenin and p‐ *β*‐catenin) and hedgehog (Bmi‐1) signaling factors (each group, n = 3). l) The mechanistic role of Notch‐1 in mediating the MB‐Drug effect is confirmed by Notch‐1 siRNA treatment for 36 h, as evidenced by the knock‐down of Notch 1 protein with preservation of Cleaved Notch‐1 and HES‐1 expression to each corresponding level of scrambled siRNA treatment as shown by the Western blot (each group, n = 3). Data are presented as mean ± S.E.M. Significance was determined by Student's t‐test analysis in (e), (j), (k) and one‐way ANOVA with Tukey's post‐hoc test in (g)–(i), (l). *p < 0.05, **p < 0.01, and ***p < 0.001 versus no treatment or between lined groups.

The concept that cancer cells can target their own type was employed to produce CaNVs. Human OC cells (SKOV3) were subjected to a series of filtering steps, thereby repeating cell membrane destruction and self‐assembly to generate nano‐size vesicles (CaNV).^[^ ^17a^ ^]^ Then, the MB‐Drug was loaded to CaNVs by using the electroporation procedure (**Figure** **2a**), which maintained the size (≈60 nm) of CaNV, as examined by transmission electron microscopy (TEM) (Figure 2b) and dynamic light scattering (DLS) (Figure 2c). Results from the MALDI‐TOF/TOF analysis confirmed the presence of Gossypol and phenformin in nanovesicles (NVs) post loading (Figure 2d). Treatment by the CaNV+MB‐Drug maintained the viability of a normal tissueoid up to the level of the no‐treatment group in contrast to the marked reduction in the viability in the OC tissueoid, indicating cancer‐specific cytotoxicity (Figure 2e). These results were supported by the OC patient cell (PAX8^+^)‐specific uptake of CaNV+MB‐Drug compared to no co‐localization observed with normal patient cells in the co‐culture of OC and normal cells (Figure 2f).

As the CaNV and MB‐Drug were merged, a synergistic anti‐cancer effect was exerted, as evidenced by the most significant reduction in the viability in OC tissueoid post CaNV+MB‐Drug treatment among the test groups (Figure 2g). This was supported by matched results from OC patient cells (Figure 2h). In contrast to the MB‐Drug treatment, the OC cell‐specific toxic effect was only observed in CaNV+MB‐Drug treatment, as normal patient cells maintained the viability to the level of the no‐treatment group. The results present a critical advantage of OC targeting effect by CaNV with reduced side effects to normal cells when the MB‐Drug is transported. The detrimental effects of CaNV on both OC and normal samples were likely driven by the Notch‐1 pathway signaling with consequent pro‐cell death effects,^[^ ^23^ ^]^ as determined in the following. These results were aligned with comparisons of the ATP level and NADH/NAD+ ratio among the test groups (Figure 2i), indicating the preserved anti‐metabolic action of MB‐Drug, even when carried by CaNV.

With respect to insight into anti‐cancer signaling of CaNV+MB‐Drug (Figure 2j), the expression of Notch‐1 downstream factors (cleaved Noth‐1 and HES‐1) significantly increased, while Notch‐1 expression remained unchanged, indicating Notch‐1 pathway as a major mechanism to drive the CaNV+MB‐Drug effect. Changes in the protein expression of Wnt and Hedgehog signaling factors were not significant upon CaNV+MB‐Drug treatment (Figure 2k). The mechanistic role of Notch‐1 was double confirmed by suppressing Notch‐1 expression using siRNA (Figure 2l), resulting in visible decreases in the expression of Notch‐1 downstream proteins (Cleaved Notch‐1 and HES‐1) upon CaNV+MB‐Drug treatment. The scrambled siRNA treatment exerted intact effect of CaNV+MB‐Drug, as shown by the significant increase in the expression of downstream factors with the maintenance of Notch‐1 expression to match that of the no treatment level.

### 2.4 Systemic Cancer Response by Xenografting

**Figure 3:**
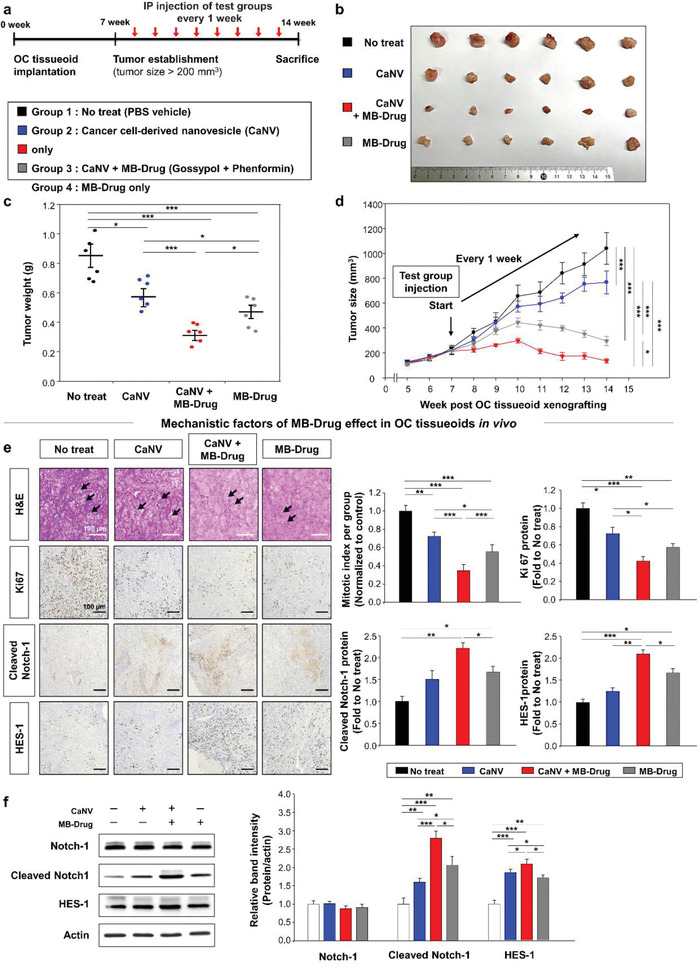
Xenografting OC tissueoid as a new systemic model to cross‐validate in vivo anti‐cancer effects of MB‐Drug. a) At week seven post OC tissueoid xenografting into ischemic hindlimbs of nude mice, tumor growth over 200 mm^3^ in volume was established. Then, test groups (no treatment– PBS vehicle control, CaNVs, CaNVs+MB‐Drug, MB‐Drug) (6 mice per group) were injected intraperitoneally every week until sacrifice. Tumor tissues were harvested at week 14. Anti‐cancer effects of test groups were determined in terms of b) optical size imaging with a ruler, c) tumor weight (gram: g), and d) quantitative analyses of progressive changes in tumor size (mm^3^). e) The mitotic index, proliferation (Ki67), and Notch‐1 factor expression (cleaved Notch‐1 and HES‐1) were analyzed quantitatively with immune‐histological staining (arrows in H&E images: apoptotic cells), f) followed by Western blot analyses of Notch signaling proteins for crosschecking. Data are presented as mean ± S.E.M. Significance was determined by one‐way ANOVA with Tukey's post‐hoc test in (c)–(f). *p < 0.05, **p < 0.01, and ***p < 0.001 versus No treat or between lined groups.

As a living body is considered as the best systemic platform for cancer growth, the OC tissueoid was xenografted into ischaemic hindlimbs in nude mice for seven weeks until tumor growth (>200 mm^3^) was established (**Figure** **3a**). Subsequently, the test drugs were injected intraperitoneally (IP) every week for the next seven weeks. Progressive changes in the tumor size (Figure 3b) and weight (Figure 3c) of each group were monitored until the end of the 14‐week post‐implantation period (Figure 3d). The results cross‐validated the most effective anti‐cancer effects of CaNV+MB‐Drug among the test groups. In agreement with the in vitro results, the use of CaNV or MB‐Drug likewise suppressed OC growth compared to no treatment. These anti‐cancer effects were confirmed by the same trends found in the mitotic index and proliferation among test groups (Figure 3e). The mechanistic involvement of Notch‐1 signaling in the CaNV+MB‐Drug effect was confirmed in this in vivo model, as the protein expression of cleaved Notch‐1 and HES‐1 significantly increased in the immunostaining analyses (Figure 3e) and Western blots (Figure 3f). As the in vitro results, the Notch‐1 expression was not altered by the test group treatments, and the use of CaNV or MB‐Drug likewise relied on Notch‐1 signaling to exert the anti‐OC effects.

### 2.5 Alignment between OC Tissueoid Responses and Clinical Outcomes

**Figure 4:**
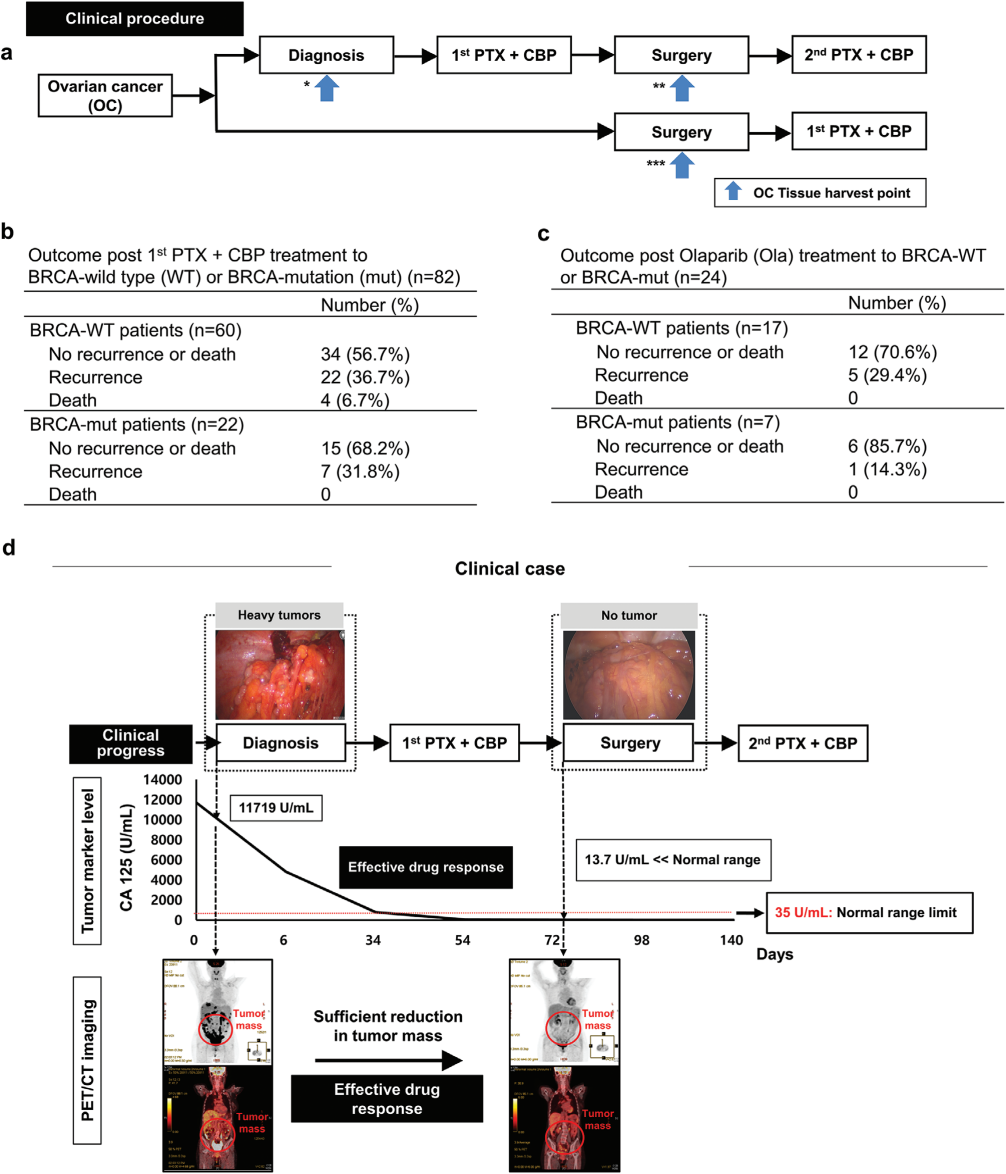
Aligned drug responses between OC tissueoid and patient tissue in‐clinic. a) During clinical progress in OC treatment, tissues were obtained from diagnosis (*) and surgery in‐between PTX+CBP (**) treatment for the drug‐first group (top) or surgery before the first PTX+CBP (***) administration for the surgery‐first group (bottom). b) In alignment with the tissuoid responses, the first PTX+CBP therapy exhibits anti‐cancer effects (>50%) on BRCA‐WT and BRCA‐mut groups (n = 82). However, significant recurrence rates (>30%) indicate the need to use another drug. c) Hence, Ola was administered to each group as maintenance therapy after PTX+CBP, resulting in a more effective tumor reduction (14.3%) in the BRCA‐mut group compared to the BRCA‐WT group (29.4%). d) As an example of alignment with the OC tissueoid response, a clinical case is shown post first PTX+CBP therapy. The effective drug response is evidenced by the significantly lowered level of the standard tumor marker (CA 125: 13.7 U mL^−1^) below the normal limit (35 U mL^−1^), which was verified by PET/CT imaging. (Red circle: tumor mass).

The test drug responses were compared with clinical outcomes by tracking OC patients for the past two years. OC tissues were obtained through biopsy (n = 82) when patients underwent surgery either before or in‐between PTX+CBP treatments (**Figure** **4a** and Table S1, Supporting Information). In alignment with the OC tissueoid responses (Figure 1f), the PTX+CBP treatment exerted anti‐cancer effects with no recurrence or death in more than 50% of the patients, regardless of the BRCA genetic status (Figure 4b). Notably, the Ola treatment on the BRCA‐mut group effectively reduced the recurrence rate to 14.3% compared to 31.8% of the PTX+CBP treatment (Figure 4c). This result is significant in targeting the BRCA‐mut group upon comparison of the reduction degree of recurrence decrease from 36.7% (PTX+CBP) to 29.8% (Ola) in the BRCA‐WT. The same conclusion was drawn when the improvement degrees of no recurrence or death from PTX+CBP to Ola treatment were compared between BRCA‐WT (56.7→70.6%) and BRCA‐mut (68.2→85.7%) groups. An impactful deliverable of the data analyses lies in the fact that the drug type‐specific efficiency and BRCA mutation‐dependent drug efficiency from the clinical outcomes can be predicted by the in vitro responses of OC tissueoids.

When the response of the OC tissueoid from one of the donor patients was compared as a representative example (Figure 1f), the clinical outcome of the same patient was clearly aligned, in that the PTX+CBP treatment progressively reduced the CA‐125 level from 11 719 at diagnosis to 13.7 U mL^−1^ before surgery (Figure 4d). The result is highly significant considering the normal upper limit (35 U mL^−1^) and is supported by the marked reduction of tumor mass upon comparison of PET/CT images at diagnosis and post‐PTX+CBP treatment. Another example of result alignment regarding a poor drug response was demonstrated by exhibiting the no‐treatment level of OC tissueoid viability from one of the patient's post PTX+CBP treatment (Figure S3a, Supporting Information). Indeed, the clinical treatment of PTX+CBP did not succeed to reduce the CA‐125 level (769 U mL^−1^) of this donor patient, as supported by no visible reduction of the tumor mass between diagnosis and before surgery, visible in the PET/CT images (Figure S3b, Supporting Information). These results represent a meaningful start to expedite new drug development by predicting clinical efficacy and efficiency in the in vitro tissueoid model as a breakthrough platform.

## 3 Discussion

In efforts spanning over the past thirty‐years, researchers have increasingly realized that an animal model is no longer reliable for the preclinical screening of anti‐cancer drugs due to inevitable gaps with clinical settings. The best approach to follow the current paradigm of patient‐specific and cancer‐specific therapy is to use patient‐derivatives, leading to the ongoing development of promising technologies, such as next‐generation sequencing, organ‐on‐a‐chip engineering, and organoid technology.^[^ ^24^ ^]^ Despite significant progress with impactful contributions, these technologies focus on readouts from the specific signature of genes, cell, structure, and biomechanical environment in some degree of combination. Hence, the current study suggests a novel approach to culture intact patient tissues in a 3D perfusible chip (tissueoid), such that the combination of possible signatures can be carried out at a single test platform, significantly improving the patient‐specificity for anti‐cancer drug screening.

This study employed the self‐homing nano‐targeting strategy to improve cancer‐specific therapeutic effects by minimizing side effects on normal cells. The concept was developed considering the nature of growth of the cancer mass based on massive reciprocal cell‐cell interactions. The use of CaNV potentiated this strategy by eliminating functional characteristics of cancer cells, such that only the key interactions among the cell membranes were preserved. The results suggest two unprecedented advantages of this approach: i) the “Trojan horse” effect to invade neighbor cancer cells by carrying anti‐cancer drugs and ii) Notch‐1 signaling from the CaNV membrane to induce cell death.^[^ ^23^ ^]^ The successful outcomes of using the OC SKOV3 cell line to produce CaNVs provide a foundation to use autologous OC cells in the following study. In this way, the self‐homing capability can be improved to target OC in the donor patients specifically.

OC was chosen for this study owing to the stable supply of patient tissues and test drugs in addition to the clinical significance. OC is a leading cause of gynecologic‐related mortality with high rates of recurrence and eventual resistance to chemotherapy. Despite the scientific progress over the last two decades, the therapeutic options for OC treatment are still limited, resulting in poor survival rates. Hence, as new drugs under pre/clinical studies, MB‐Drug and Ola were included for comparison with the general chemotherapy (PTX+CBP) for drug type‐specific efficiency, BRCA mutation‐dependent drug efficiency, and metabolism inhibition‐based anti‐cancer effects. As a pioneering approach, these drug effects were cross‐validated by the in vitro model of the OC tissueoid with the clinical outcomes or the new systemic model of in vivo cancer responses.

The clinical outcomes of Ola and PTX+CBP treatments from 104 patients were available to compare with the OC tissueoid results, the preclinical study status of MB‐Drug motivated the development of the new in vivo model was used for comparison instead of clinical outcomes. The in vivo model established two major observations regarding the cancer environment: i) hindlimb ischaemia represents the state of insufficient oxygen supply with massive cancer growth, and ii) aggressive blood vessel growth surrounding the tumor provides oxygen and nutrients to growing cancer cells. Further, the perfusion connection of the microchannel network with in‐growing host vessels sets up routes for cancer cells and soluble factors to move into the circulatory system, as shown in our previous studies.^[^ ^3^, ^25^ ^]^ In this manner, cancer propagation into other organs through the circulatory track can be monitored while examining systemic side effects of the drug when injected through IP, as used in this study, or even intravenous (IV) routes.

In the in vivo model, the repeated injection of MB‐Drug reduced the tumor weight and size significantly compared to No treat (Figure 3b–d). The synergistic action of cargo and drug was seen in the results of CaNV+MB‐Drug, suggesting a key role of CaNV in helping MB‐Drug to stay longer in OC tissuoid under implantation by prolonging metabolic clearance. OC cell‐specific targeting of CaNV was also validated through a series of in vitro models (Figure 2e,f), and thus, in vivo investigation of distribution and metabolism was needed to confirm the feasibility and advantages of OC tissueoid. However, a proper control to investigate this aspect could not be found due to the following reasons. First, since OC tissue itself exhibited promotive effects on angiogenesis as evidenced by the high‐level expression of Notch‐1 signaling molecules^[^ ^1^ ^]^ (Figure 3e,f), the control to remove OC tissue in the 3D perfusable chip could not set up the same base level of blood vessel formation to enable perfusion connection with host circulation. This was critical to investigate the effect of OC tissue absence on CaNV clearance by shorting the stay time in the 3D chip because angiogenesis‐mediated perfusion connection between channel network and host vessels served as a pivotal route for CaNV to reach the target site.

Second, the control to leave the ischemic limb without implanting OC tissue and 3D chip could not also set the proper level of perfusion connection with body circulation for CaNV delivery due to the role of 3D microchannel network chip in promoting the connection as seen in our previous study.^[^ ^2^ ^]^ Third, non‐cancer ovarian tissues were not provided by the clinic side due to ethical issues. Forth, the CaNV could be compared with normal ovarian cell‐derived NVs upon implantation of OC tissueoid, but enough population of normal cells to inject NVs into mice repeatedly (160 µg mL^−1^ for 8 weeks) could not be achieved due to the limited proliferation capability (maximum passage 2–3). Fifth, stem cell‐derived NVs could be considered, but as reported previously,^[^ ^3^ ^]^ their nature of homing to inflammatory sites (e.g., ischemic hindlimb) generated another variable to limit fair comparison of tumor targeting with the CaNV.

This study applied convergent technologies to demonstrate breakthrough concepts including the OC tissueoid, CaNV, new drugs, in vivo model of systemic cancer response, and to cross‐check with clinical outcomes. Promising results at the present step suggest the feasibility to reach more successful levels towards clinical translation. A wider range of patient‐specific signatures (e.g., age, pathologic background, family history, etc.) in addition to BRCA mutation must be included to improve the precision in the drug choice selection. Moreover, as human samples were xenografted into nude mice in this study, the roles of immune responses in the in vivo model of systemic cancer response must be investigated. Matching autologous CaNVs with the donor OC specimen was limited due to the size and heterogeneity of patient specimens. The cross‐validation of MB‐Drug effects between the OC tissueoid and clinical outcomes must be verified when the drug undergoes clinical trials in the future.

## 4 Experimental Section

### Ovarian Cancer (OC) Patient Tissues and Cells

Patient tissue specimens were obtained at Yonsei Cancer Center (Seoul, Korea) under approval by the Institutional Review Board (IRB) of Yonsei University College of Medicine (IRB No. 4‐2018‐0342) in accordance with the guidelines. Target patients were selected by reviewing their medical records including age, CA‐125 levels at diagnosis, FIGO stage^[^ ^15^ ^]^ (i.e., malignant stage based on cancer size and progress), BRCA gene status, histological subtype, chemotherapy regimen, surgery type, and outcomes (i.e., recurrence and death). The study included only the patients with advanced‐stage epithelial OC from the first clinic and thus, excluded the groups with low potential malignancies (e.g., non‐epithelial and borderline epithelial OC)^[^ ^26^ ^]^ or under re‐entrance of chemotherapy due to recurrence. Recurrence was defined as the appearance of gross disease in imaging (e.g., CT scan) or clinical examination (e.g., palpable mass). Women at high risk of breast and/or OC(s) were tested to determine mutation(s) of BRCA1 and BRCA2 genes to select the best treatment.^[^ ^27^ ^]^ Participant consent was received after informing each patient about the research‐only use of clinical data.

Fresh OC or normal tissues were harvested immediately (< 1 h) at the time of surgery through biopsy and kept on ice (Figure 4a). Only OC dominant tissues were used by excluding specimens with small, scattered tumors and/or scar appearance (e.g., necrotic or fibrotic). To harvest cells, patient tissues were washed with PBS; cut into pieces (≈5 mm), and incubated with collagenase type I (2 mg mL^−1^; Gibco, Carlsbad, CA, USA) and DNase I (10 µg mL^−1^; Sigma‐Aldrich, St. Louis, MO, USA) in RPMI1640 (Gibco, Carlsbad, CA, USA) for 1 h at 37 °C by mixing occasionally. After the reaction mixture was filtered through a cell strainer (70 µm pore diameter, Falcon, Durham, NC, USA), cells were collected by centrifuging at 1300 rpm for 3 min and cultured until their use in the experiments.

### 3D Tissue Culture in Gelatin Hydrogel Chip with Channel Network (Tissueoid)

The tissueoid was produced by culturing patient tissue pieces directly in a microchannel network hydrogel. Tissue pieces, each of 1 mm diameter, were obtained by punching the patient tissue and embedded into a gelatin hydrogel together with a thread of PNIPAM fibers (Mn ≈ 85 000, Sigma–Aldrich, St. Louis, MO, USA) to make channels post melting and washing, as described in previous studies.^[^ ^3^, ^16^ ^]^ A custom‐built spinning device was operated with a 45% PNIPAM/MeOH solution to generate the fiber thread,^[^ ^28^ ^]^ and the diameter of PNIPAM fiber was controlled by adjusting spinning speed (2500–2800 rpm). The PNIPAM fibers at a density of 11.45 ± 3.13 µg mm^−3^ were placed in the PDMS mold, and a silicone tube was placed to connect with the fibers at the inlet and outlet sides. In this way, a closed perfusion system was generated to enable the flow of culture media from the inlet to the outlet of silicone tube by passing through the fiber‐generated channel network. Next, patient tissue pieces were mixed with a gelatin/mTG solution (9: 1 ratio, final concentration 5% w/v), and the mixture gel solution was poured onto the fibers, followed by a cross‐linking reaction at 37 °C. The embedded fibers were dissolved from the mTG hydrogel by sol–gel transition of PNIPAM at room temperature with perfusing PBS. The tissueoid was subjected to 3D culturing with perfusing media at a continuous flow rate of 20 µL min^−1^ through the channel network.

### OC Patient Characteristics of Tissueoid

To determine whether the OC tissueoid preserves the corresponding patient characteristics, OC patient tumor tissues and corresponding tissueoids were subjected to immunohistochemistry of OC markers (p53 and PAX8) and hematoxylin and eosin (H&E) staining. After slicing samples into 4 µm sections, the sections were fixed in 10% formalin, embedded in paraffin, deparaffinized three times with xylene, rehydrated with alcohol, heated using a microwave, and boiled twice for 6 min in 10 mm citrate buffer (pH 6.0). Each section was then blocked by treatment with 3% hydrogen peroxide and 4% peptone casein solution for 15 min. Next, the sections were incubated with p53 and PAX8 antibodies (Novus Biologicals, CO, USA) at room temperature for 40 min and then with horseradish peroxidase‐conjugated secondary antibodies (rabbit or mouse; Dako, Glostrup, Denmark), followed by optical imaging (Leica DMi8, Leica Microsystems, Wetzlar, Germany).

### Viability of OC Tissueoid to Anti‐Cancer Drugs

The general chemotherapy drugs included PTX, CBP, and Ola (Sigma‐Aldrich, St. Louis, MO, USA). The MB‐drug was a combination of gossypol and phenformin (Sigma‐Aldrich, St. Louis, MO, USA). In vitro viability of OC tissueoids upon treatment of anticancer drugs was determined at week one post perfusion culture both with and without drugs. The live/dead assay was conducted by loading calcein‐AM and ethidium homodimer‐1 (Thermo Fisher Scientific, Waltham, MA, USA) to perfusion media for 30 min, followed by confocal imaging (LSM 980, Zeiss, Oberkochen, Germany) and quantitative analysis.

### Western Blot of OC Tissueoid to Anti‐Cancer Drugs

Drug responses between OC patient tissues from the clinic and artificial cancer tumoroids were compared by Western blot analyses. Proteins were extracted by OC tissues by removing fats on ice and homogenizing using an ice‐cold lysis buffer [150 mm NaCl, 0.5% Triton‐X 100, 50 mm Tris‐HCl (pH 7.4), 20 mm ethylene glycol tetra‐acetic acid, 1 mm dithiothreitol (DTT), 1 mm Na_3_VO_4_ and protease inhibitors, 1 mm phenylmethyl sulfonylfluoride (PMSF), and ethylenediaminetetraacetic acid (EDTA)‐free cocktail tablet] with periodical vortexing for 30 min. The lysates were centrifuged at 14 000 g for 15 min at 4 °C. The protein supernatants were collected and stored at 70 °C until use. The corresponding protein concentration of each sample was determined using BCA protein assay kit (Pierce, Rockford, IL, USA).

Proteins were separated by running through 8–12% SDS‐PAGE gel and electrotransferred onto a nitrocellulose membrane, followed by blocking in 5% non‐fat dry milk/TBST (Tris‐buffered saline buffer containing 0.1% Tween‐20) for 1 h at room temperature. The membranes were incubated with primary antibodies of apoptosis markers PARP, cleaved caspase‐3, cyclin D1, and PCNA; 1: 1000, Cell signaling, MA, USA], ERK signaling markers [phosphor (p)‐ERK and ERK; 1: 1000, Cell signaling, MA, USA], and AKT signaling markers [p‐AKT and AKT; 1: 500, Abcam, Cambridge, UK], BRCA1 (1:500, Abcam, Cambridge, UK), and *β*‐actin (1: 2000, Santa Cruz Biotechnology, TX, USA) with dilution in TBST, overnight at 4 °C. Blots were rinsed three times with TBST at 10 min intervals and incubated with horseradish peroxidase‐conjugated secondary antibodies (rabbit, mouse, or goat; Dako) in TBST for 1 h at room temperature. The band intensities were visualized using an enhanced chemiluminescent (ECL) detection kit (Bio‐Rad, CA, USA) and quantified using LAS‐3000 Image Analyzer (Fujifilm, Tokyo, Japan).

### Cancer Energy Metabolism

The anti‐cancer effects of the MB‐Drug were characterized by determining i) mitochondrial activity (MitoTracker Red CM‐H_2_Xros selective probe, Thermo Fisher, Waltham, MA, USA),^[^ ^29^ ^]^ ii) ATP level (Celliter‐Glo Luminescent Cell Viability Assay kit, Promega, Durham, NC, USA),^[^ ^30^ ^]^ and iii) NADH/NAD+ ratio (NADH/NAD+ quantitation colorimetric kit, BioVision, CA, USA)^[^ ^31^ ^]^ according to the manufacturer's instructions. (i,ii) Patient tissue‐derived cells were seeded in a 96‐well plate (1 × 10^5^ cells/well) and treated both with and without MB‐Drug (gossypol 1 µm and phenformin 10 µm) for 48 h.

i) After the cells were treated with CellTiter‐Glo and lysed, the number of metabolically active cells was determined by measuring the ATP level with colorimetric reading using a microplate reader (BioTek, Seoul, South Korea).
ii) After cell lysis using a buffer provided in the kit, half of the lysate was heated to 60  °C for 30 min to decompose NAD+ to NAD while keeping NADH intact. Both NAD and NADH were reacted with NAD cycling enzyme, followed by a colorimetric reading at 450 nm using a microplate reader (BioTek) to determine the total amount of NAD. With the other half of cell lysate, the amount of NADH was quantified using an NADH standard curve. Each NAD and NADH amounts were normalized to the corresponding protein amount (mg) post determination using BCA protein assay kit (Pierce, Rockford, IL, USA). Finally, the ratio of NADH to NAD+ (total NAD – NADH) was calculated.
iii) Cells were treated with the MitoTracker (100 nm) for 30 min at 37 °C post MB‐drug treatment in a 4 well chamber slide (1 × 10^5^ cells/well) for 24 h, followed by inverted microscopic imaging (Leica DMi8, Leica Microsystems, Wetzlar, Germany).

### CaNV Production with Loading of Anti‐Cancer Energy Metabolism Drug (MB‐Drug)

OC cells (SKOV3) were propagated up to approximately 80% confluency and collected in suspension by detachment with 0.25% trypsin/EDTA (Gibco, NY, USA). Then, as reported previously,^[^ ^17a^ ^]^ cell suspensions in PBS (1 × 10^7^ cells mL^−1^) were subjected to a series of filtering steps by decreasing pore sizes of polycarbonate membrane filters (Whatman, Maidstone, UK) from 10 to 5 µm and then to 0.4 µm using an extruder kit (Avanti Polar Lipids, AL, USA), thereby producing CaNVs. After CaNVs were collected by centrifuging at 13 000 g for 30 min, intact NV formation around a 100 nm diameter range was verified, as reported in a previous study.^[^ ^17a^ ^]^ Subsequently, MB‐drugs were loaded into CaNVs by using the Neon electroporation system (Invitrogen, Carlsbad, USA).^[^ ^32^ ^]^ Briefly, CaNV (1 mg mL^−1^) was mixed with MB‐Drugs (gossypol 10 µm and phenformin 100 µm) in 10 µL of resuspension buffer, followed by electroporation (1400 V, 2 ms, and 2 pulse length). Unreacted MB‐drugs were removed through tubing dialysis (MWCO = 20 kDa, Spectrum Lab, Greece) for 24 h and then collected by centrifugation at 15 000 g for 30 min. The sizes and morphologies of CaNV test groups were characterized by TEM (JEM‐F200, JEOL, Tokyo, Japan) and a DLS spectrophotometer (DLS, DLS‐7000, Otsuka Electronics Ltd., Tokyo, Japan), respectively. Drug loading was confirmed by matrix‐assisted laser desorption ionization mass spectrometer (MALDI‐TOF/TOF 5800 system, AB SCIEX, Framingham, MA, USA) analysis of CaNV+MB‐Drug.^[^ ^33^ ^]^ Briefly, each sample (0.5 µL) was spotted on Opti‐TOF 384‐Well Insert (AB SCIEX, Framingham, MA, USA) with 50% acetonitrile (0.5 µL) in 0.1% trifluoroacetic acid (Merck, Darmstadt, Germany), followed by drying at room temperature. Data acquisition was conducted by processing MALDI‐TOF spectra by using Data Explorer software (version 4.11, AB SCIEX, Framingham, MA, USA).

### In Vitro Anti‐Cancer Effects of CaNVs and MB‐Drugs on OC Tissueoids

The anti‐cancer effects of CaNV+MB‐Drug (20 µg mL^−1^) were first examined by determining the viability of OC cells or tissueoid using a live/dead assay as described above, followed by quantitative image analysis using ImageJ (NIH, Stapleton, NY, USA).

Further, self‐homing nano‐targeting of CaNVs was examined by determining OC cell‐specific internalization of CaNVs by immunocytochemistry. CaNV+MB‐drug (20 µg mL^−1^) were labeled with a DiD fluorescent dye (Vybrant DiD Cell‐Labeling Solution, Thermo Fisher Scientific, Waltham, MA, USA) and treated to a mixture of OC and normal patient cells in culture on a 4‐well chamber slide (1 × 10^4^ cells/well). Cells were then fixed in 95% methanol for 10 min at −20 °C and rinsed with PBS containing 0.1% Tween 20 (PBST, pH 7.4), followed by permeabilization with 0.2% Triton X‐100 in PBS for 5 min. After washing three times with PBST, cells were blocked for 2 h in PBST containing 5% bovine serum albumin. Samples were treated with PAX8 (OC marker) primary antibody (1: 100, Novus Biologicals, CO, USA) in PBST with 1% BSA overnight at 4 °C. After washing three times with PBST, samples were treated with FITC‐conjugated anti‐rabbit IgG secondary antibody (1: 1000) in PBST with 1% BSA at room temperature for 1 h. Cells were then counter‐stained with 4′,6‐diamidino‐2‐phenylindole (DAPI, Vector Labs, Burlingame, CA, USA) and rinsed with PBST, followed by confocal imaging of colocalization between CaNV+Drug and OC cells (Leica DMi8, Leica Microsystems, Wetzlar, Germany).

Western blot analysis was conducted on OC tissueoids upon MB‐Drug treatment to determine signaling factors of the drug mechanism. The representative markers of general anti‐cancer mechanisms included Notch (Notch‐1 and cleaved Notch‐1, Cell signaling, MA, USA), Wnt (*β*‐catenin and phosphor (p)‐*β*‐catenin, Cell signaling, MA, USA), and Hedgehog (Bmi‐1, Cell signaling, MA, USA). Moreover, a mechanistic role of Notch‐1 in the drug effect was confirmed by knock‐down with siRNA transfection into OC cells (1 × 10^5^ cell mL^−1^) before MB‐Drug treatment. Notch‐1 siRNA (25 nm) was transfected into OC cells with lipofectamine RNAiMAX (Invitrogen, Waltham, MA, USA) reagent according to the manufacturer's instructions. The siRNA sequence was: (forward) 5’ –GUG UGA AUC CAA CCC UUG U‐3’ and (reverse) 5’ –ACA AGG GUU GGA UUC ACA C‐3’ (Bioneer, Seoul, South Korea).

### Xenografting OC Tissueoids into Mice as a New In Vivo Model of Systemic Cancer Response

All animal experiments were approved by the Institutional Animal Care and Use Committee of Yonsei University College of Medicine (authorization number: 2019‐0296). Six‐week‐old female BALB/c nude mice (total 24, n = 6 mice per group) were purchased from Orient Bio (Seoul, South Korea), followed by quarantine under specific pathogen‐free conditions with a 12 h light/ dark cycle. The model of OC tissueoid implantation into ischemic hindlimbs of mice was used to establish a new model of systemic cancer response, as reported in previous studies.^[^ ^3^, ^25^ ^]^ The OC tissueoid was labeled with a fluorescent dye solution (Vybrant DiD Cell‐Labeling Solution, Thermo Fisher Scientific, Waltham, MA, USA) in culture medium (1:200 dilution) at 37 °C with 5% CO_2_ for 1 h. The upper and lower points of the femoral artery were ligated using a 4‐0 silk suture (Ethicon, Somerville, NJ, USA), and the femoral artery was dissociated and separated from the vein, followed by implantation of OC tissueoid into hindlimb muscle. Cancer propagation from the implantation site before and after OC tissueoid harvest was visualized using an in vivo imaging system (IVIS, PerkinElmer, Waltham, MA, USA).

### In Vivo Tissueoid Responses to Anti‐Cancer Drugs

Anti‐cancer effects of test groups (PBS vehicle control, CaNV, CaNV+MB‐Drug, and MB‐drug) were determined by analyzing inhibition of tumor growth and proliferation. IP injection of test groups in PBS (1 mL) was initiated post tumor growth at least 200 mm^3^ at week 7 post OC tissueoid implantation and carried out every week until week 14. Tumor volume was measured with digital calipers and calculated according to the following formula: volume (mm^3^) = 0.5 × a × b^2^ [Diameters: a (longest) ┴ b (shortest)] At day 14‐week post‐implantation, tumor tissues were harvested for further analyses after euthanizing mice with CO_2_ gas. The tissues were then fixed in 10% formaldehyde, embedded in paraffin, and cut into 4 µm sections. Then, they were subjected to H&E staining, followed by optical microscopy imaging (Leica DMi8, Leica Microsystems). Samples were deparaffinized, rehydrated, and treated with primary antibodies of Notch‐1 pathway (cleaved notch‐1 and Hes‐1, Cell signaling) and proliferation (Ki67, Abcam, Cambridge, UK) at room temperature for 40 min and then with horseradish peroxidase‐conjugated secondary antibodies (rabbit or mouse; Dako), followed by optical imaging (Leica DMi8, Leica Microsystems). Protein expression of signaling markers including Notch‐1 pathway (cleaved notch‐1 and Hes‐1 Cell signaling) and proliferation (Ki67, Abcam) was determined using tumor tissues by Western blotting, as described previously.

### Crosscheck with Clinical Data

The tumor stage of each patient was screened by CT and PET/CT scanning before surgery and determined by histological analysis with a pathological report post tumor resection surgery. As the primary OC treatment, all patients underwent surgical removal of cancer mass^[^ ^34^ ^]^ (Figure 4a), followed by a standard surgical procedure including total hysterectomy, bilateral salpingo‐oophorectomy, retroperitoneal lymphadenectomy, or omentectomy. The patients then underwent chemotherapy with PTX+CBP treatment before and after surgery (Figure 4a). Some patients with sufficient responses after PTX+CBP treatment received Ola, which inhibits a PARP enzyme and thereby, repairs DNA as a means to maintain the disease‐free status.^[^ ^14^, ^35^ ^]^ Drug responses were determined by examining the level of CA‐125 with PET/CT imaging. The following data were extracted and analyzed from patient medical records to crosscheck the validity of experimental data (Figure 4 and Figure S3, Supporting Information), which includes the age, CA‐125 level at diagnosis and before DS, FIGO stage, BRCA mutation, tumor histology, surgery type, chemotherapy regimen, and outcome (e.g., recurrence and death).

### Statistical Analysis

The normality of data distribution was determined using the Shapiro–Wilk test. All normally distributed data were expressed as the means ± standard error of the mean (SEM) while non‐normally distributed data were expressed as median (minimum‐maximum). Categorical data were described by frequencies and percentages (%). All clinical data were represented as median (minimum‐maximum). All in vitro and in vivo data were presented as means ± SEM from at least three independent experiments. Single comparison between two groups was conducted by a two‐tailed Student's t‐test. One‐way analysis of variance (ANOVA) with Tukey's significant difference post‐hoc test was applied for multiple comparisons. Values of * *p* < 0.05, ** *p* < 0.01, and *** *p* < 0.001 were considered statistically significant. The relevant sample size (n number) of each experiment was denoted in the corresponding figure legend. All statistical analyses were carried out using Excel and SigmaPlot V.12.0 (Systat Software Inc., San Jose, CA, USA).

## Conflict of Interest

The authors declare no conflict of interest.

## Author Contributions

H.‐J.Y., Y.S.C., and Y.J.L. contributed equally to this work as co‐first authors. J.‐Y.L., S.K., and H.‐J.S. (Lead) are listed as the co‐corresponding authors, considering their significant contributions. H.‐J.S. designed and directed the entire study. J.‐Y.L. and S.K. oversaw the clinical aspects of the study. H.‐J.Y., Y.S.C., and Y.J.L. led the experiments, analyzed the data, and prepared the figures and tables. S.E.Y. and H.S.K collaborated on the in vitro studies, and S.B. assisted in the animal studies. H.‐J.S. drafted the manuscript with assistance from H.‐J.Y and Y.S.C.

## Supporting information

Supporting Information

## Data Availability

Research data are not shared.
