# Supplementary material for: Cancer Patient Tissueoid with Self‐Homing Nano‐Targeting of Metabolic Inhibitor
Source: Adv Sci (Weinh). 2021 Oct 18;8(22):2102640. doi: 10.1002/advs.202102640 (PMC8596099; doi:10.1002/advs.202102640)
Supplement: Supplementary file 1 — Supporting Information [file ADVS-8-2102640-s001.pdf]

© 2021 Wiley-VCH GmbH

ADVANCED SCIENCE
Open Access

Supporting Information

for *Adv. Sci.,* DOI: 10.1002/advs.202102640

Cancer patient tissueoid with self-homing nano-targeting of metabolic inhibitor

Hyo-Jin Yoon, Young Shin Chung, Yong Jae Lee, Seung Eun Yu, Sewoom Baek, Hye-Seon Kim, Sang Wun Kim, Jung-Yun Lee, Sunghoon Kim*, and Hak-Joon Sung**

Supplementary Figures

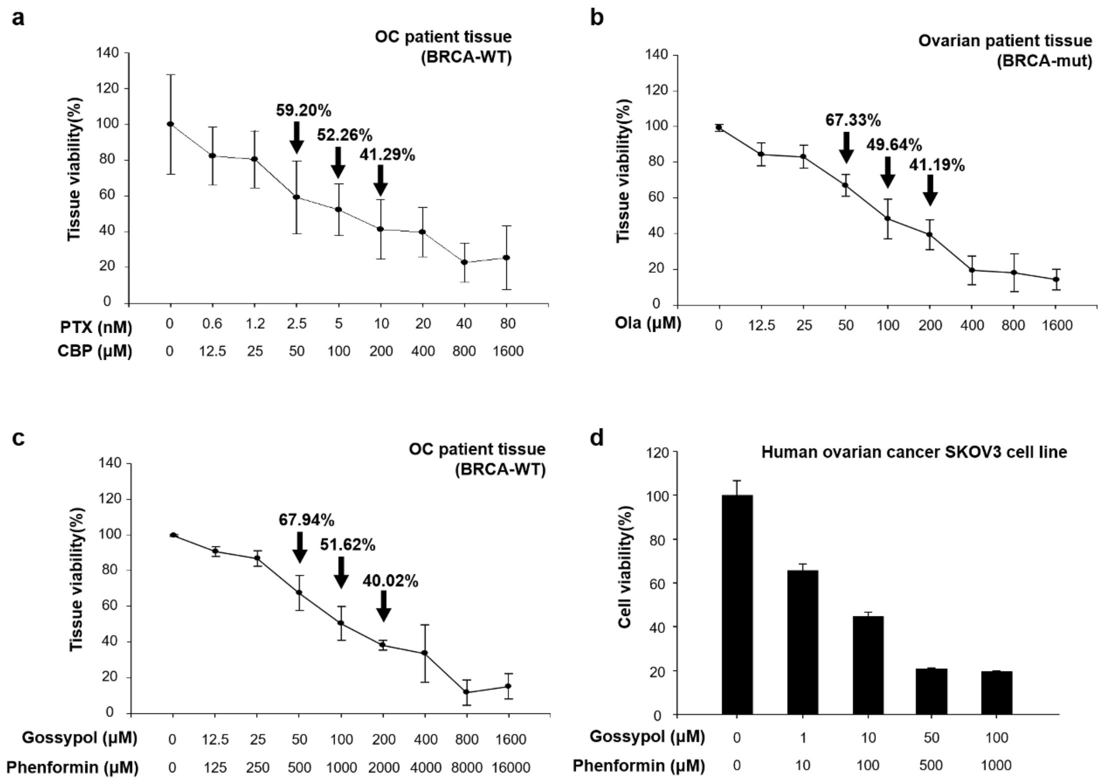

Figure S1. Half maximal inhibitory concentration (IC 50) of target drugs. IC 50 of each test drug was determined by analysing the viability of OC patient derivatives by varying the drug concentrations as follows. **(a)** Paclitaxel (PTX) + carboplatin (CBP) on BRCA-WT tissue; **(b)** Olaparib (Ola) on BRCA-mut tissue; and MB-Drugs (gossypol + phenformin) on **(c)** BRCA-WT tissue, and **(d)** human ovarian cancer SKOV3 cell line.

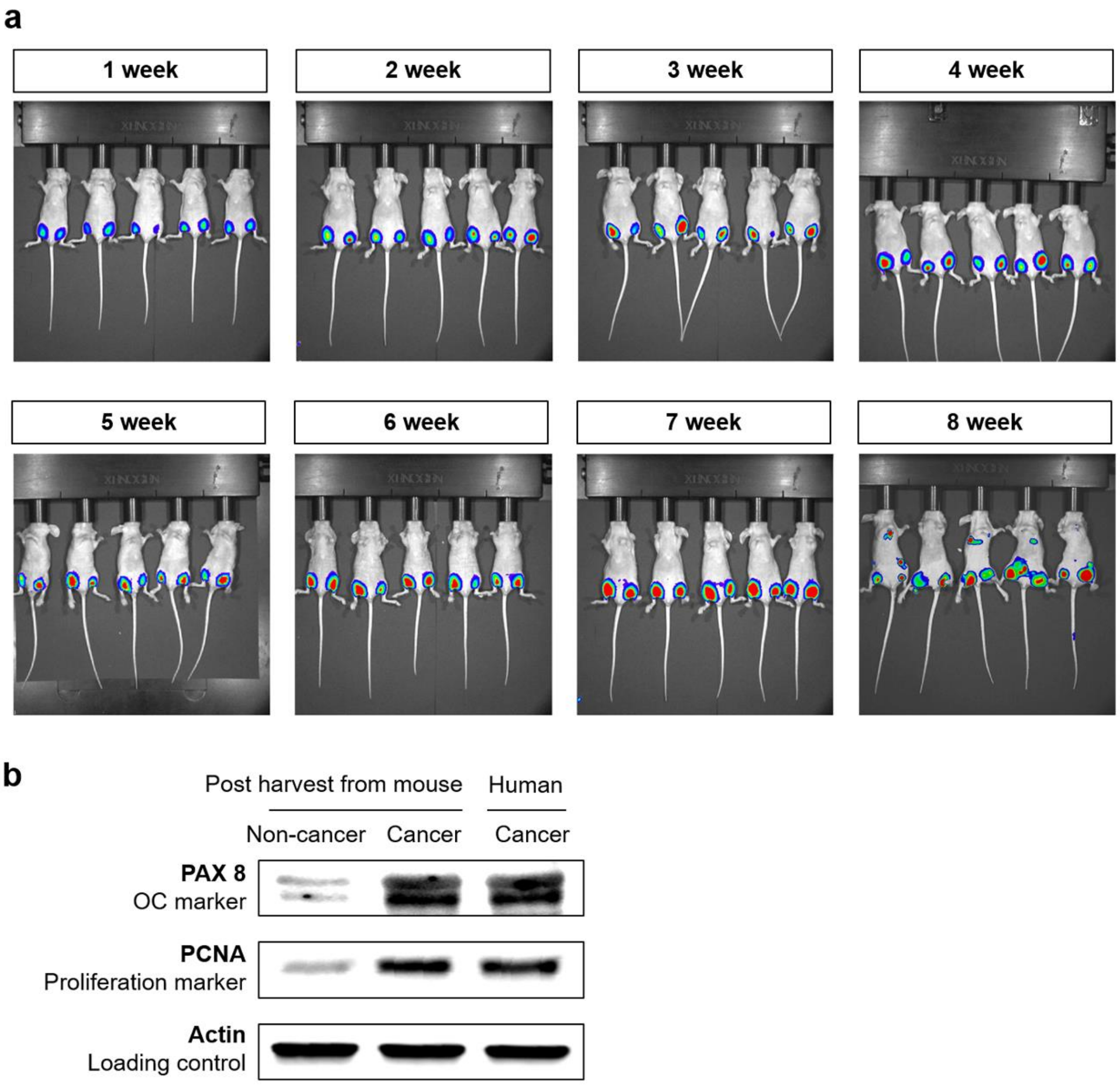

Figure S2. *In vivo* cancer growth post xenografting of OC tissueoids. (a) Tissue samples were labeled with DiD fluorescence dye, followed by generation and implantation of OC tissueoids in ischemic hindlimbs of nude mice. Cancer growth in the implanted sites is monitored by IVIS imaging every week. **(b)** Preservation of human OC characteristics post implantation into nude mice is confirmed by analysing expression of PAX8 (human OC marker) and PCNA (proliferation marker) through the Western blot (Actin: loading control).

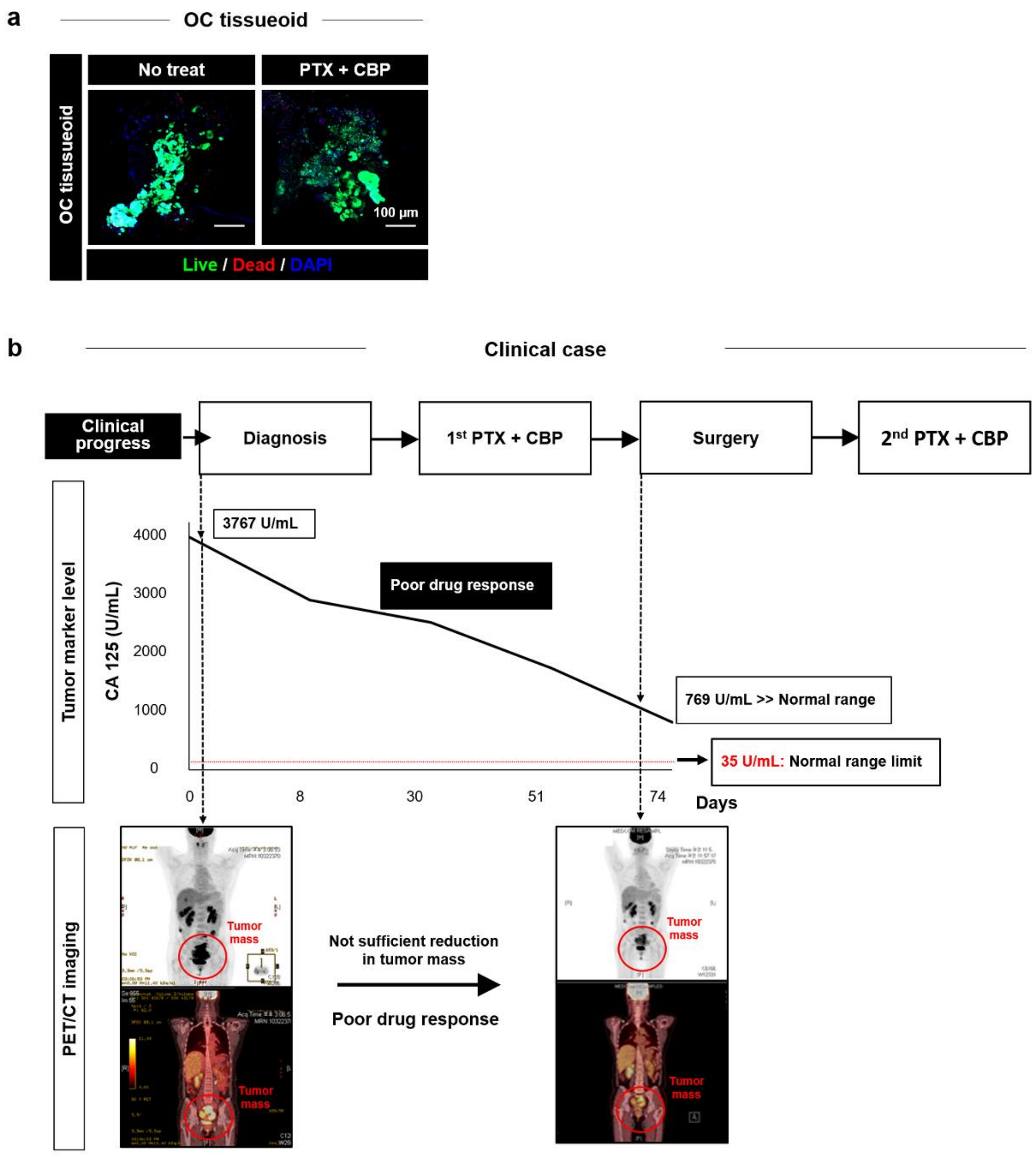

Figure S3. Aligned case of poor drug response between OC tissueoid and clinic. (a) As an indication of poor drug response, the intact viability of OC tissueoid is preserved even after PTX+CBP treatment, as shown by confocal imaging. **(b)** In alignment with the tissueoid response, a clinical case confirms the poor drug response until the end of first PTX+CBP therapy, because the level of standard tumour marker (CA 125: 769 U/mL) is higher than the normal limit (35 U/mL). The result is double-checked by PET/CT imaging. (Red circle: tumour mass).

Supplementary Data Table 1. Patient characteristics (n=104)

Diagnosis	Median (– range)
Age at diagnosis	57.5 (37.0–78.0) years
CA-125 level at diagnosis	1735.6 (75.2–14838.2) U/mL
**FIGO stage**	**Number (%)**
III	45 (43.3%)
IV	59 (56.7%)
**Histological subtype**	**Number (%)**
High-grade serous carcinoma	86 (82.7%)
Low-grade serous carcinoma	2 (1.9%)
Clear cell	6 (5.8%)
Mucinous	3 (2.9%)
Endometrioid	7 (6.7%)
**Chemotherapy regimen**	**Number (%)**
PTX + CBP	104 (100%)
**Surgery type**	**Number (%)**
Primary debulking surgery	53 (51.0%)
Interval debulking surgery	51 (49.0%)

CA-125, cancer antigen-125; FIGO, International Federation of Gynecology and

Obstetrics; PTX + CBP, paclitaxel + carboplatin.
